# The London Hospital—I

**Published:** 1893-06-03

**Authors:** 


### The London Hospital—I.

*(By our Special Commissioner.)*

During the last twenty-five years, as the writer knows from personal experience, in this country a revolution has taken place in hospital methods, construction, and nursing. This fact is not sufficiently appreciated by inexperienced enthusiasts, who in large numbers are, we are glad to say, beginning to interest themselves in hospital work. It is one thing for a hospital committee, with a virgin site, and £100,000 to spend in new buildings, to conduct a great medical institution, replete as it can be made in such circumstances with every modern improvement and appliance. It is quite another thing to be in the position of a committee like that of the London Hospital, which has to provide for the conduct of the largest hospital in this country, portions of which were built from 100 to 150 years ago, and the whole of which has grown by degrees, and so necessarily contains patent defects and shortcomings which should be wholly absent in a new building, though, we regret to say, some of our most recent hospitals contain even graver structural defects than can be met with to-day in any part of the London Hospital. A great institution like the London should successfully appeal to the sympathy and support of the great of our day and generation: great, that is, in the sense of their responsibility for the conduct and management of great businesses, huge manufacturing or trading concerns, which entail a vast amount of administrative ability, apart altogether from the mere provision of suitable buildings, capable employés, and the requisite funds. It is not possible for an ordinary person, however intelligent and willing he may be, to visit the London Hospital, to go through its wards and offices, and to come away with anything like an intelligent, practical, and true idea of the enormous work done, or of the exceptional efficiency of that work. The very vastness of the institution, and the complexity of its many departments and extensive ramifications are a distinct disadvantage in many ways. We spent eight hours in the London Hospital quite recently, and devoted most of that time to a careful inspection of the institution; but, although our inspection was made with an exceptional knowledge of the buildings, a thorough understanding of the work, and of each and all of the many departments into which that work is divided, we found it impossible to compass the whole, although we worked hard to accomplish this result. If this be the fact, and it is an undoubted fact, then in all sober earnestness we would urge those who have not the technical training, the knowledge, or the opportunity to form a judgment of the excellence of this vast work for ministering to the sufferings of the poor of East London, to accept our testimony, and to believe that, taken as a whole, and in detail, there is no establishment of any kind, devoted to any purpose in this country to-day, which can claim to be more efficient or to better fulfil the objects and purposes for which it was established than the London Hospital. The time has arrived when it is necessary for the committee of the London to make its quinquennial appeal for £40,000 per annum for the next five years. Speaking from fulness of knowledge, and the most earnest conviction, we venture to say that every person who has a little money to give to charitable objects should, in the first instance, give to the London Hospital, and that the people of this country, and especially the inhabitants of London, should not rest content, nay, should not go to the country for their annual holiday, unless they have assured themselves that by one combined effort this £40,000 a-year for five years has been placed to the credit of Messrs. Robarts, Lubbock, and Co., 15, Lombard Street, London, E.C., the bankers of this noble charity.

### An Appeal to Editors.

We have said that the London Hospital ought to continuously have the sympathy and support of the head of every great institution and business in this country. This is so because no one else is so well able as these gentlemen to appreciate the wonderful character, extreme value, and marvellous efficiency of the vast establishment in the Whitechapel Road. Every able administrator appreciates good work when he sees it, and we believe that it would be really profitable if every one of the great men we have in our mind would make it their business to give up a couple of days in order that they may spend them in systematically examining into and following out the work of the London Hospital. We would especially appeal to our brother editors in the metropolitan Press, and would ask them to tell off the best of their special correspondents, and to send them into the London Hospital to spend a couple of days within its wards, and then find space for the publication of their reports. If they would do this, and if, in the course of the second day, the editors themselves would go to the London and get their correspondents to show them what they have discovered in regard to the work there, the Press would be so impressed and awakened that we should never more have to complain of irresponsible and false criticism concerning any hospital in the future, so far as the London Press is concerned. Why should this suggestion not be entertained, and promptly acted upon, seeing that "the copy" that might be supplied would be of supreme interest to readers of all classes? June 11th next has been fixed as Hospital Sunday in London, and we would beg our brother editors throughout the metropolitan Press to make this the occasion for adopting the suggestion here made, and thus rendering a public service to Londoners which it would be difficult to exaggerate or to otherwise accomplish. The benefits which the London Hospital confers are not confined to the 10,000 in-patients who occupy the 780 beds in the course of the year, or to the 120,000 out-patients who receive medical aid and medicine. Those benefits are equally conferred upon each and all of the many employés of all classes, from the doctors to the humblest members of the staff, who daily labour with a conscientiousness and an *esprit* which would do credit to the most efficient and best disciplined army which the world has ever seen. Our great voluntary hospitals, indeed, want less criticism and more cash, but their value would be immeasurably increased if it were possible to set the fashion amongst the leisured classes, who have money to spare, to resort to the hospitals, or, at any rate, to send representatives at regular intervals to visit and report upon these institutions, in order that they might be kept in remembrance not only of the work done, but of the woes and wearinesses of the sufferers they contain, who, without our hospitals, must undergo an immensity of suffering—now happily avoided, and often be prematurely cut off from life itself. Will not our brother-editors bestir themselves now, in this year of financial panic and exceptional stress, and, by giving a trial to the suggestions here made, do more in a week to help the hospitals, and, through the hospitals, to help not only the poor and suffering, but the leisured and indolent, than has been yet done at all in this nineteenth century?

### The General Impressions of a Day's Tour.

We will now try to give some idea of what an expert sees and feels during an inspection of a great hospital like the London. Remembering what hospitals were, and seeing what they are to-day, wonder and thankfulness compete for the mastery of his mind—wonder at the enormous developments which are apparent in every direction—the improved mechanical appliances, the added comforts, the glorious increase in the amount of air and sunlight and decorative aids everywhere present, the growth in the number of nurses, and the enormous improvement in the morale, the appearance, the skill, and ability of these devoted women. Surely such object-lessons must produce wonder, and they cannot fail to inculcate thankfulness too! A pessimist in a hospital ward under present conditions must, despite himself, be made cheerful, and even hopeful. Everywhere the evidence of self-denial, and the result of that self-denial and patient training is exhibited in the skill and tenderness, the forethought and considerate kindness of all who have to do with the care and cure of the sick, which must impress and stimulate the most sluggish of our race. There is no escape from the evidence of all this, because that evidence abounds in all directions. Flowers and plants, tasty bed coverings, pictures and artistically-coloured walls, little helps to comfort, like the special pillows which the Jewish patients are encouraged to bring with them, or the bed-tables which the prone patients are supplied with. Here, an ingenious contrivance to screen a patient from the draught, which hygienic considerations necessitate; there, a little private room, sheltered from the noise and the bustle of the ward in order to give the sickest of all the patients the maximum of opportunity to regain the lost strength. All this must cheer and even surprise a stranger, and would make the hospital worker of thirty years ago rub his eyes in astonishment, whilst he marvelled how it was possible to have introduced all these great improvements and still to maintain the hospital by voluntary contributions. There is one other and probably the principal cause for all the change for the better, which one meets everywhere. It is the introduction of an army of devoted women, who are highly educated and elaborately trained, and the cheeriness of whose presence and their genuine enthusiasm for the work brighten everything they come in contact with, and so convert a house of suffering into a home of cheerfulness, and cheerfulness is, after all, the high road to recovery. Of course, there are wards in the London Hospital, like Rowsell in the basement, which structurally are very imperfect, and there is again the erysipelas ward, which almost necessarily leaves something to desire in the same direction; but when you speak to the sister or the nurses in charge of these wards their faces brighten, and they will at once give you half-a-dozen good reasons why Rowsell or the erysipelas wards are, after all, the very best wards in the hospital in which to work, because they spend their lives there, and they know by actual experience, and who should know better, that this is the case? Formerly, the attendants in wards of this description were not always of the highest class. To-day, however, it is not too much to say that there is not a member of the staff who would not cheerfully go to any ward, however humble, or however imperfect, and contentedly and devotedly labour there, not only with courage, but with a happy assurance that, on the whole, there was no other place where good work could be better or more usefully done. We are but relating facts as we found them at the London Hospital on the day of our visit, and nothing we could write would surely carry conviction as to the condition of the internal arrangements of this great institution better than they do. It may appear to answer the purpose of a few individuals to abuse the internal administration of the London Hospital, but we make bold to say that there is not a governor, nay, there is not an impartial man or woman in the country, who, having visited and studied the inner workings of this great charity, but would boldly declare that it is a pleasure to see how completely the whole machine moves, because every portion of the machinery, and every individual member of the staff is happily conscious of the fact that these excellent results can only be attained by individual devotion to duty, and the habit of a cheerful spirit, and true devotion to the work in hand for that work's sake, and that alone. In the matter of the food, too, everything is done to secure that each patient shall have the particular portion which will give him or her the greatest satisfaction, and that every patient, too, shall be served in an appetising manner. No wonder then the visitor finds that, despite the suffering, there is everywhere evidence of genuine happiness and contentment within the walls of the London Hospital.

### A Hint to the Rich.

Inside the wards, too, the sisters and nurses vie with each other in the endeavour to make each ward more attractive than any other ward by cultivating flowers and plants and ferns and other greenery for its beautification and refreshment. In one ward we noticed that an intelligent citizen, Mr. R. Thorpe, gives £6 a-year in order to keep one bed bright with flowers and suitable plants. How many rich people have a superabundance of such things, and why should they not instruct their gardener to send regularly every month a waggon-load of suitable plants to one of the hospitals, and so thin out much that is useless to them, whilst it would prove a delight to many a sick man and woman, whose recovery might even be hastened by such surroundings!

*(To be continued.)*

